# Perinatal depression and breastfeeding: a longitudinal investigation of the bidirectional relationship in Pakistan

**DOI:** 10.1007/s00737-026-01722-1

**Published:** 2026-06-13

**Authors:** Sarah C. Haight, Lisa M. Bates, Esther O. Chung, Amanda Collins, Allison Frost, Aparna G. Kachoria, Kaitlin Shartle, Siham Sikander, Joanna Maselko

**Affiliations:** 1 Department of Epidemiology and Biostatistics, University of Georgia, Athens, USA; 2 Department of Epidemiology, Columbia University, New York, USA; 3 RTI International, Research Triangle Park, USA; 4 Department of Epidemiology, University of North Carolina at Chapel Hill, Chapel Hill, USA; 5 Carolina Population Center, University of North Carolina at Chapel Hill, Chapel Hill, USA; 6 Department of Maternal and Child Health, University of North Carolina at Chapel Hill, Chapel Hill, USA; 7 Sanford School of Public Policy, Duke University, Durham, USA; 8 Department of Primary Care & Mental Health, University of Liverpool, Liverpool, UK; 9 Pakistan Institute of Living and Learning, Karachi, Pakistan

**Keywords:** Depression, Breastfeeding, Pregnancy, Postpartum, LMICs

## Abstract

**Purpose:**

The relationship between perinatal depression and breastfeeding is complicated and reciprocal. While breastfeeding may protect against postpartum depression, perinatal depression can also reduce the likelihood of breastfeeding. Using longitudinal data from Pakistan, we examine the effect of (1) breastfeeding on postpartum depression, stratified by prenatal depression and (2) perinatal depression on breastfeeding.

**Methods:**

Data were drawn from the Bachpan study in Pakistan at five timepoints: pregnancy, delivery, 3-, 6-, and 12-months postpartum. Breastfeeding (any and exclusive [EBF]) was assessed using 24-hour recall; depression was measured with the Structured Clinical Interview for DSM-5 (SCID). Marginal structural models accounted for the time-varying relationship between depression and breastfeeding to estimate risk ratios (RR) and 95% confidence intervals (CI).

**Results:**

Among 1,014 postpartum individuals, weighted prevalence of any breastfeeding was 73.6% at delivery, 93.1% at 3-months, 86.1% at 6-months, and 73.0% at 12-months. EBF at 6-months was low (8.7%). Depression prevalence was 25.1% during the third trimester, 13.1% at 3-months, 10.8% at 6-months, and 15.7% at 12-months. Any breastfeeding was associated with a lower risk of postpartum depression (RR: 0.77; 95% CI: 0.51–1.03), though the CI included the null. In turn, perinatal depression reduced the likelihood of any breastfeeding (RR: 0.92; 95% CI: 0.84–0.99). EBF was similarly associated with a lower risk of postpartum depression (RR: 0.94; 95% CI: 0.68–1.20), though the CI also included the null. When stratified by prenatal depression, point estimates suggest that EBF may have a stronger association with lower depression risk among those without prenatal depression compared to those with prenatal depression (RR: 0.77 vs. RR: 1.03), though both CIs included the null.

**Conclusions:**

Results support a complex bidirectional relationship between breastfeeding and perinatal depression, where depression reduces breastfeeding likelihood and any breastfeeding shows possible protective associations with depression, though the benefits of EBF may differ by prenatal depression status.

## Background

Breastfeeding offers well-documented benefits for both postpartum people and their infants, including lower risk of cancer and cardiovascular disease for breastfeeding parents (Ebina and Kashiwakura 2012; González-Jiménez et al. 2014; Tschiderer et al. 2021) and reduced risk of sickness, obesity, and high blood pressure for infants (Duijts et al. 2010; Brion et al. 2011; Horta et al. 2015). Informed by these benefits, exclusive breastfeeding (EBF) for the first six months postpartum is recommended by the World Health Organization (WHO 2018). At six months, the introduction of complementary foods along with continued breastfeeding up to two years is recommended. Despite these recommendations, breastfeeding practices are influenced by numerous factors. Rollins et al. (2016) conceptualized these influences as operating across the structural (e.g. sociocultural norms), setting (e.g. availability of breastfeeding counseling and education), and individual (e.g. mental health) levels (Rollins et al. 2016). At the individual level, depression can play a critical role in breastfeeding, but this relationship is complex given its bio-social nature and bidirectionality.

Breastfeeding and perinatal depression are linked through both biological and psychosocial pathways. Biologically, breastfeeding stimulates the release of oxytocin and prolactin, hormones that influence stress reactivity (Stuebe et al. 2013; Whitley et al. 2020; Bruce et al. 2025). Simultaneously, the social context of breastfeeding, shaped by cultural expectations and personal experiences, can impact maternal well-being. (Rollins et al. 2016). Successfully meeting breastfeeding recommendations and expectations may foster feelings of efficacy and satisfaction, while struggling to do so because of pain, supply, or lack of support, may contribute to stress, guilt, and shame (Dennis 1999). Even minimal breastfeeding may reflect significant emotional effort in the face of difficulty, highlighting the importance of examining any breastfeeding, not just exclusivity in relation to depression.

The relationship between breastfeeding and depression is also bidirectional. Perinatal depression can lead to reduced breastfeeding intention and early cessation (Gagliardi et al. 2012; Jain et al. 2014; Rahman et al. 2016; Coo et al. 2020; Yahya et al. 2021), while breastfeeding can protect from postpartum depression (PPD) onset or aid in recovery from depression (Seimyr et al. 2004; Mezzacappa and Endicott 2007; Hamdan and Tamim 2011; Figueiredo et al. 2013; Hahn-Holbrook et al. 2013; Toledo et al. 2022). Current research on this bidirectionality is inconsistent, highlighted by three systematic reviews (Dennis and McQueen 2007; Dias and Figueiredo 2015; Butler et al. 2021). This cyclical relationship is difficult to study with cross-sectional designs, which dominate the existing literature and limit the ability to establish temporality or account for pre-existing depression and time-varying confounders. Longitudinal studies have been conducted, but mostly in high-income countries like the U.S. and U.K. (Henderson et al. 2003; Pippins et al. 2006; Cooke et al. 2007; McCarter-Spaulding and Horowitz 2007; Dennis and McQueen 2007; Fairlie et al. 2009; Bogen et al. 2010; Hamdan and Tamim 2012; Ystrom 2012; Figueiredo et al. 2014; Dias and Figueiredo 2015; Ahlqvist-Björkroth et al. 2016; Castro et al. 2017; Haga et al. 2018; Alimi et al. 2022; Yuen et al. 2022).

The few existing studies in low- and middle- income countries (LMIC) reflect the bidirectional relationship with some reporting breastfeeding to be protective for PPD and others noting that PPD reduces the likelihood of breastfeeding (Nazel and Nosseir 1994; Hasselmann et al. 2008; Rahman et al. 2016; Yusuff et al. 2016a; Tuthill et al. 2017). However, a recent systematic review reported that the majority of these studies were cross-sectional and lack data from South Asia (Lubis et al. 2024). While one studied this relationship prospectively, it was only investigated in one direction (the impact of depressive symptoms on EBF duration) (Rahman et al. 2016). To date, only three cross-sectional studies have investigated the relationship between breastfeeding and PPD in Pakistan, all concluding either that depression reduces the likelihood of breastfeeding (Taj and Sikander 2003) or that breastfeeding was associated with a reduced risk of PPD (Rehman and Atiq 2016; Shah and Lonergan 2017). Understanding the bidirectional relationship between breastfeeding and depression in Pakistan requires consideration of the local breastfeeding context. While breastfeeding initiation is relatively high, fewer than half of infants are exclusively breastfed for the first 6 months in Pakistan (National Institute of Population Studies Islamabad Pakistan 2019). Further, within the first three days of life, over three-quarters of infants are given something other than breastmilk such as honey, ghutti (herbal mixture), dates, water, or tea (National Institute of Population Studies Islamabad Pakistan 2019). These practices reflect traditional beliefs that prelacteal feeds create a bond between the individual providing it and the infant and protect against feeding colostrum, which is sometimes viewed as old or harmful milk (Zakar et al. 2018; Asim et al. 2020). However, recent qualitative work suggests that these perceptions may be shifting, with community health workers reporting increased awareness of colostrum’s benefits and the importance of exclusive breastfeeding (Singletary and Farooqi 2024) Given this cultural context where breastfeeding is normative, but exclusivity is not, the biological and psychosocial pathways linking breastfeeding and depression may operate differently than in other LMIC settings where practices and norms differ.

This study addresses existing gaps by using longitudinal data from rural Pakistan to investigate the bidirectionality of the relationship between breastfeeding and depression by documenting the effect of (1) breastfeeding on PPD, stratified by prenatal depression and (2) perinatal depression (depression spanning pregnancy to 12-months postpartum) on breastfeeding. For the former, we hypothesize that breastfeeding will reduce the risk of PPD, but that among those with prenatal depression, the protective effect will be weaker, or reversed, relative to those without prenatal depression. For the latter, we hypothesize that perinatal depression will be associated with a reduced likelihood of any and exclusive breastfeeding.

## Materials and methods

### Data source

Data are from the Bachpan Cohort located in rural Pakistan. The cohort originated from

a stratified, cluster-randomized controlled trial of a maternal depression intervention in 40 village

clusters, across one sub-district of the Rawalpindi district. For the trial, eligible participants included

individuals aged $\:\ge\:$18 in their third trimester of pregnancy who were registered with local community health workers. For every depressed participant (Patient Health Questionnaire-9 [PHQ-9] scores ≥10)

enrolled in the trial, a non-depressed participant (PHQ-9 < 10) was enrolled as well, creating a cohort with equal numbers of participants with and without prenatal depression at the beginning of data collection. Informed consent was obtained from all individual participants included in the study (Sikander et al. 2019). Assessments were done during pregnancy, and 3-month, 6-month, 12-month, 2-years, 3-years, 4-years, 6-years, and 8-years postpartum. Further information about the parent study is available elsewhere (Maselko et al. 2020). While the Bachpan study has baseline (pregnancy) data for 1,154 participants, the current analysis only utilized data from those with any follow-up data (*n* = 1,014). To capture breastfeeding consistent with existing WHO recommendations, data from baseline, 3-, 6-, and 12-month waves were used. All waves were combined in final models to assess the directionality of the relationship between breastfeeding and depression, but not the impact of time itself.

## Measures

Within the Bachpan Cohort, in addition to PHQ-9 scores, perinatal depression was also assessed using the Structured Clinical Interview for DSM Disorders (SCID) current major depressive episode (Spitzer et al. 1992) during the third trimester of pregnancy (baseline) and at 3-, 6-, and 12-month postpartum. The SCID is a semi-structured clinical interview used to diagnose psychiatric disorders. Assessors determined whether each participant met the criteria for a current major depressive episode at the time of the interview. The Urdu version of the SCID has validated (Rahman et al. 2003) and is widely used for perinatal populations in South Asia (Gorman et al. 2004; Nast et al. 2013).

Breastfeeding at delivery, 3-, 6-, and 12-months postpartum were obtained by asking: “What has the child had in the last 24 hours?” with check all that apply options including breast milk, *ghutti*, herbal water, water, tea, formula milk, animal milk, semi solid food, and solid food. Information on breastfeeding at delivery was asked at the 3-month assessment where individuals were asked to think retrospectively to what their baby had within 24 h of being born. If they indicated that the child consumed *any* breast milk in the last 24 h, they were considered to have breastfed at that time. Any indication of breastfeeding was used for the main analysis in response to the concept that breastfeeding is a biosocial concept (Butler et al. 2021) and any amount of it may play a role in the cyclical relationship with depression. We also examined the relationship between EBF and depression up to 6 months postpartum (per WHO recommendations), and vice versa. EBF was considered *only* breastmilk in the past 24 h at the current wave and all previous 24 h recalls, with the exception of one-time use of *ghutti* or herbal water at delivery due to its use as a cultural practice, rather than a feeding method (Zakar et al. 2018; Asim et al. 2020). See Appendix I for more details.

Potential confounders at baseline were: trial arm (intervention, control, non-depressed), maternal age, years of education, socioeconomic status (SES) (measured via assets [electronics, appliances, transportation, home materials, and facilities] and standardized using a polychoric principal components analysis and summed) (Kolenikov and Angeles 2009; Maselko et al. 2018), number of living children, household structure (nuclear, non-nuclear), history of ever experiencing physical, psychological, or sexual intimate partner violence (IPV; WHO Violence Against Women Instrument) (WHO 2005), and social support (Multi-dimensional Scale of Perceived Social Support [MSPSS]) (Zimet et al. 1988, 1990). An additional confounder at 3-months was the observance of *chilla*, the traditional postpartum practice of receiving rest and support for 40 days. The assessor that collected the data was also considered a confounder and was adjusted for at the respective wave (baseline, 3-months, 6-months, and 12-months) using a categorical variable based on years of experience.

## Analysis

For descriptive statistics, all percentages, means, and standard deviations were weighted to account for the overrepresentation of depressed participants and the cluster sampling design of the Bachpan study to generate a sample that is representative of the local population. Specifically, participants that were non-depressed at baseline were upweighted to account for their sub-sampling during study recruitment (approximately 1 in 3) and depressed participants (who were all invited to participate in the RCT) received a weight of one (Maselko et al. 2018). All N’s are unweighted. We modeled the relationship between breastfeeding (any and EBF) and depression in two different directions: (1) breastfeeding on PPD, stratified by prenatal depression and (2) depression on breastfeeding. We first used multiple imputation by chained equations (Azur et al. 2011; Wulff and Jeppesen 2017) to account for loss-to-follow up and item-level missingness in the key variables (breastfeeding, depression, and confounding variables) among respondents that were present for at least one follow-up wave (*n* = 1,014). Twenty imputations were generated using baseline confounders and variables associated with missingness (*p* < 0.1) at each time of follow-up. The latter included variables related to the household (number of people per room, maternal grandparent living in home, husband’s work status), health (disability, illness, blood pressure), other psycho-social factors (instrumental and emotional social support, risk-taking behaviors, activation, maternal self-efficacy, duration of *chilla*), and the child’s development. Multiple imputation resulted in complete data for 1,014 participants at all four timepoints used for the regression models.

**Fig. 1:**
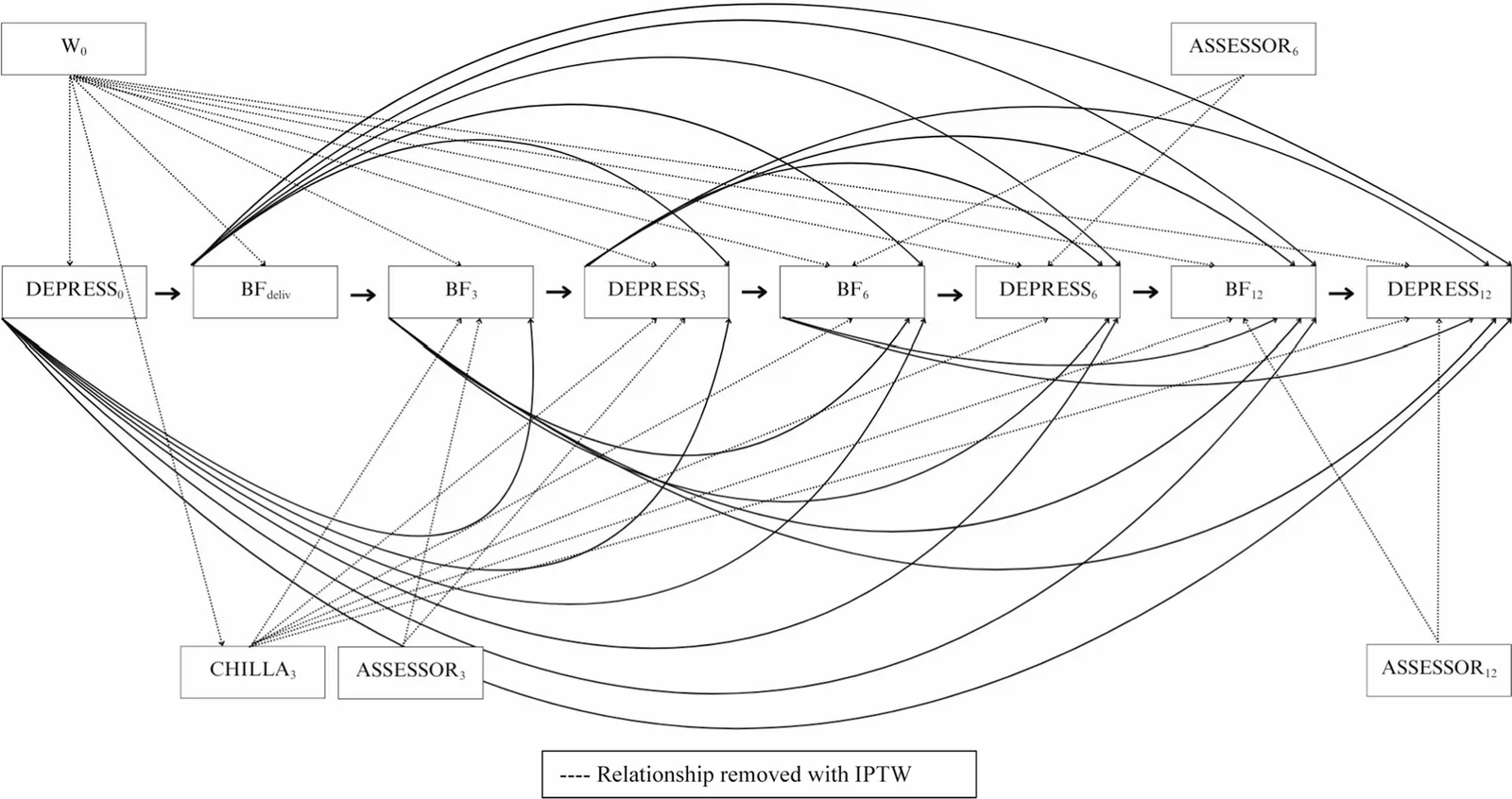
Directed acyclic graph for the relationship between *breastfeeding*^*a*^ *and depression*^*b*^ and from third trimester of pregnancy (t=0) to 12-months postpartum (t=12)

**Fig. 2:**
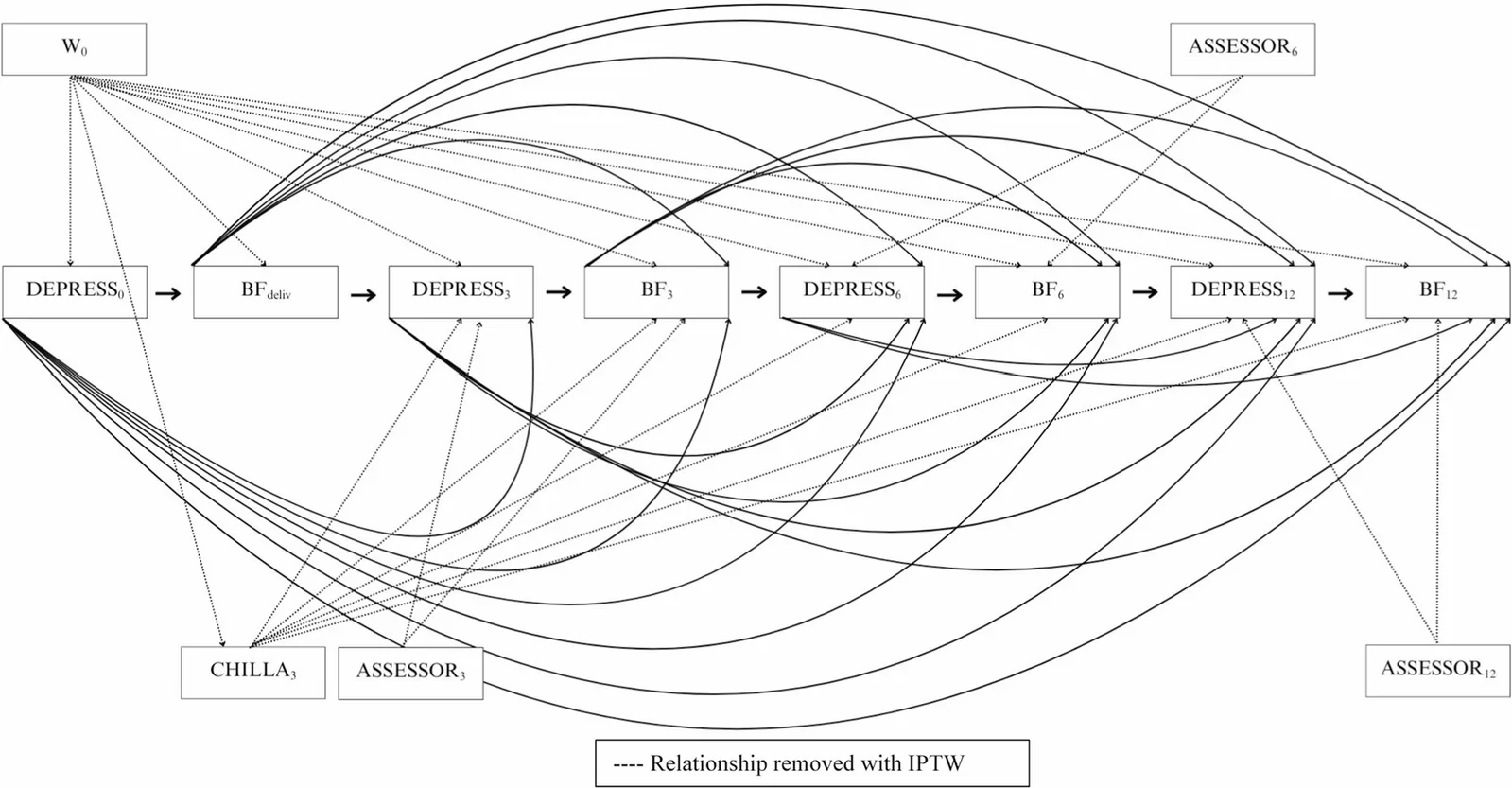
Directed acyclic graph for the relationship between *depression*^*a*^ *and breastfeeding*^*b*^ from third trimester of pregnancy (t=0) to 12-months postpartum (t=12)

When studying whether breastfeeding affects depression and whether depression affects breastfeeding over time, the central methodological challenge is that these factors influence each other, making it difficult to isolate the independent effect of one on the other. Depression status may affect ability to breastfeed, which in turn may affect future depression, creating a complex web of relationships in which these time-varying variables predict future exposures and are affected by past exposures (Figs. 1 and 2). Standard regression models cannot untangle these relationships and can induce bias by adjusting for variables that are on the pathway. To address this, we utilized marginal structural models (MSMs) with inverse probability of treatment weights (IPTWs). MSMs are specifically designed to address this ‘time-varying confounding’ where a variable can simultaneously serve as a confounder, exposure, and outcome at different time points (Robins et al. 2000). IPTWs work by creating a pseudo-population in which the exposure (breastfeeding or depression, depending on the direction being examined) is independent of measured confounders, essentially balancing comparison groups as if they had been randomly assigned to breastfeed or have depression. Specifically, our IPTWs account for time-invariant confounders measured at baseline (age, education, SES, number of living children, household structure, IPV, and social support), time-varying confounders (assessor), and time-varying exposure status (depression or breastfeeding at earlier time points). By balancing these confounders across exposure groups, this approach reduces bias from time-dependent confounding and allows for estimation of the independent relationship of each variable on the other while accounting for the bidirectional relationship over time. Stabilized IPTWs for each research question were calculated as:

**Formula 1.**

$$\begin{array}{l}{IPTW}_{BF}\\=\:\frac{\mathrm{Pr}\left({BF}_{3}=1|{BF}_{deliv}\right)}{\mathrm{P}\mathrm{r}({BF}_{3}=1|{BF}_{deliv},{DEP}_{0},{W}_{0},{C}_{3}{,A}_{3}}\times\\\frac{\mathrm{Pr}\left({BF}_{6}=1|{BF}_{deliv},{BF}_{3}\:\right)}{\mathrm{P}\mathrm{r}({BF}_{6}=1|{BF}_{deliv},{BF}_{3},{DEP}_{0},{DEP}_{3},{W}_{0},{C}_{3}{,A}_{6}}\times\\\frac{\mathrm{Pr}\left({BF}_{12}=1|{BF}_{deliv},{BF}_{3},\:{BF}_{6}\right)}{\mathrm{P}\mathrm{r}({BF}_{12}=1|{BF}_{deliv},{{BF}_{3},\:{BF}_{6},DEP}_{0},{DEP}_{3},{DEP}_{6},{W}_{0},{C}_{3},{A}_{12}}\end{array}$$

**Formula 2.**

$$\begin{array}{l}{IPTW}_{DEP}\\=\:\frac{\mathrm{P}\mathrm{r}({DEP}_{0}=1)}{\mathrm{P}\mathrm{r}({DEP}_{0}=1|{W}_{0})}\times\\\frac{\mathrm{Pr}\left({DEP}_{3}=1|{DEP}_{0}\:\right)}{\mathrm{Pr}\left({DEP}_{3}=1|{DEP}_{0},{BF}_{deliv},{W}_{0},{C}_{3}{,A}_{3}\right)}\times\\\frac{\mathrm{Pr}\left({DEP}_{6}=1|{DEP}_{0},\:{DEP}_{3}\right)}{\mathrm{P}\mathrm{r}({DEP}_{6}=1|{DEP}_{0},{DEP}_{3},{BF}_{deliv},{{BF}_{3},W}_{0},{C}_{3}{,A}_{6}}\times\\\frac{\mathrm{Pr}\left({DEP}_{12}=1|{DEP}_{0},{DEP}_{3},\:{DEP}_{6}\right)}{\mathrm{P}\mathrm{r}({DEP}_{12}=1|{DEP}_{0},{{DEP}_{3},\:{DEP}_{6},BF}_{deliv},{BF}_{3},{BF}_{6},{W}_{0},{C}_{3},{A}_{12}}\end{array}$$

Time is indicated with subscripts (0 = baseline; deliv=delivery; 3 = 3-months postpartum; 6 = 6-months postpartum; and 12 = 12-months postpartum). BF is breastfeeding, DEP is depression, W is baseline confounders, C is *chilla*, and A is assessor. Because prenatal depression at baseline occurred prior to breastfeeding at delivery, unlike the other timeframes in which they were reported concurrently, depression and breastfeeding at these times were considered as confounders in Formula 1, not part of the independent/dependent variables in the final models.

Under the identification assumptions, the estimated risk ratios (RR) from the model for Formula 1 represent the marginal (average) effect of breastfeeding on subsequent depression across the postpartum period (3, 6, and 12 months), comparing those who breastfed at a given time point versus those who did not, while accounting for time-varying confounding and prior exposure history. Similarly, the estimated RRs from the model for Formula 2 represent the marginal effect of depression on subsequent breastfeeding practices across the postpartum period, comparing those with depression at a given time point versus those without, while accounting for time-varying confounding and prior exposure history. Since the EBF analysis was restricted to 6-months, we conducted a robustness check restricting the any breastfeeding analysis to 6-months to ensure differences were not simply due to the restricted time frame. All analyses were conducted using SAS 9.4 (Cary, NC). Bachpan data for the first three waves are available in the Harvard Dataverse (Gallis et al. 2024); additional data are available upon reasonable request from the senior author.

## Results

During pregnancy, participants were on average, 26.5 years old (SD: 5.4), had 8.1 years of schooling (SD: 5.5), 1.4 children (SD: 1.7), and perceived social support ranged from 1 to 7 with a mean of 4.7, indicating a moderately high level of social support (Table 1). Assets-based SES scores ranged from − 5 to 2.8, with a mean of 0.2 (SD: 0.2), meaning that our sample was slightly above the theoretical midpoint of the distribution, suggesting modest asset ownership. The majority lived with a non-nuclear family (88.0%) and did not have a history of experiencing IPV (54.2%; Table 1). At 3-months postpartum, the majority of participants had observed *chilla* (89.6%; Table 1). The weighted prevalence of any breastfeeding was 73.6% at delivery, 93.1% at 3-months, 86.1% at 6-months, and 73.0% at 12-months postpartum. The weighted prevalence of EBF was 56.1% at delivery (allowing for the inclusion of *ghutti* or herbal water), 49.3% at 3-months, and 8.7% at 6-months. The weighted prevalence of depression was 25.1% during the third trimester, 13.1% at 3-months, 10.8% at 6-months, and 15.7% at 12-months.

MSM results suggest that any breastfeeding was associated with a lower risk of PPD, an estimated 25% reduction in risk, though the CI included the null (RR: 0.77; 95% CI: 0.51–1.03; Table 2). The relationship was similar among those who were prenatally depressed (RR: 0.74; 95% CI: 0.44–1.05) and not prenatally depressed (RR: 0.80; 95% CI: 0.40–1.21), suggesting no modification by prenatal depression. In the converse direction, perinatal depression reduced the likelihood of any breastfeeding by approximately 8% (RR: 0.92; 95% CI: 0.84–0.99).

Compared to any breastfeeding, EBF showed a more attenuated association with PPD (RR: 0.94; 95% CI: 0.68–1.20) with the confidence interval including the null (Table 3). However, estimates stratified by prenatal depression status suggest potential modification, where individuals with prenatal depression showed no relationship between EBF and PPD (RR: 1.03; 95% CI: 0.71–1.34), while those without prenatal depression showed a possible protective association (RR: 0.77; 95% CI: 0.31–1.23), though both confidence intervals included the null. In the reverse direction, perinatal depression was associated with a higher likelihood of EBF, but the CI included the null (RR: 1.19; 95% CI: 0.99–1.39). Results restricting the any breastfeeding analysis to 6-months were robust indicating that the differing results between any and EBF were not due to the restricted timeframe (Appendix II).

## Discussion

In this study of 1,014 perinatal people in rural Pakistan, we found high rates of breastfeeding initiation within 24 h of delivery (73.6%) and continued breastfeeding at 3-months (93.1%), 6-months (86.1%), and 12-months postpartum (73.0%). However, only 9% were EBF at 6-months postpartum. This low prevalence aligns with previous reports of markedly low EBF country-wide (< 15%) based on Demographic Health Survey Data from 2010 to 2018 (Zong et al. 2021), but is substantially lower than more recent estimates from 2017 to 2018 alone (48%) (National Institute of Population Studies Islamabad Pakistan 2019). Our sample is situated within a rural setting, which may strongly influence breastfeeding behaviors. Model results suggest that any breastfeeding was associated with approximately 23% lower risk of PPD, regardless of prenatal depression status, although the confidence interval included the null. Conversely, perinatal depression reduced the likelihood of any breastfeeding by approximately 8%. Although attenuated, EBF showed a possible protective association with PPD, with a stronger effect observed among those without prenatal depression compared to those with prenatal depression.

Our findings contribute to the evidence of the protective effect of breastfeeding on PPD in LMIC settings. The aforementioned systematic review examining this relationship across LMICs found that breastfeeding was associated with a reduced likelihood of PPD, though effect sizes varied considerably across studies and settings (Lubis et al. 2024). Our point estimates align with this general protective pattern observed in several cohort studies (Ding et al. 2014; Yusuff et al. 2016b; Agler et al. 2021), though our confidence intervals include the null, consistent with several other studies that found non-significant associations in the expected direction (Sharifi et al. 2016; Farías-Antúnez et al. 2020). As it pertains to studies conducted specifically in Pakistan, our patterns similarly align with cross-sectional studies reporting that breastfeeding was associated with a lower prevalence of depression (Rehman and Atiq 2016; Shah and Lonergan 2017). Notably, few previous studies have employed causal inference methods like MSMs to address time-varying confounding or examined the bidirectional relationship between breastfeeding and depression simultaneously, which may explain some of the inconsistency in prior literature.

The attenuated association observed for EBF compared to any breastfeeding with PPD is potentially counterintuitive and warrants discussion. We expected stronger protective effects for EBF as it likely provides greater hormonal exposure (more frequent nursing stimulates higher and more consistent release of oxytocin and prolactin) which dampens stress reactivity and promotes maternal-infant bonding (Stuebe et al. 2013; Whitley et al. 2020; Bruce et al. 2025). However, our findings suggest that psychosocial mechanisms may be equally or more important than biological effects in this context. In rural Pakistan, where breastfeeding initiation is nearly universal (93% at 3 months in our study), but EBF rates are markedly low (9% at 6 months in our study), successfully initiating and maintaining any breastfeeding may protect mothers from social stigma, criticism from family members, and feelings of inadequacy as a mother (Dennis 1999; Rollins et al. 2016). Meeting this breastfeeding expectation, regardless of exclusivity, may confer psychological benefits through enhanced self-efficacy and social acceptance. In contrast, EBF represents a departure from traditional practices that include prelacteal feeds (Asim et al. 2020) and may be difficult to sustain without robust family support, potentially introducing stress that counteracts biological benefits.

In our investigation of whether prenatal depression status modified the effect of breastfeeding on PPD, we found evidence of modification for EBF, but not for any breastfeeding. We hypothesized that while breastfeeding may be generally protective against PPD, the demands and difficulties of breastfeeding combined with potentially altered biological responses among already-depressed individuals might diminish or even reverse its protective effects. This hypothesis was supported for EBF: those without prenatal depression showed a protective association (RR: 0.77), while those with prenatal depression showed no benefit (RR: 1.03). However, we did not observe this modification pattern for any breastfeeding, where the association was similarly protective regardless of prenatal depression status. This may suggest that the threshold of difficulty for any breastfeeding, which allows for supplementation and flexibility, remains low enough that postpartum people with depression can achieve it without the practice becoming counterproductive.

For the second research question, depression predicted a reduced likelihood of any breastfeeding, but not EBF. The former is consistent with existing work suggesting that prenatal depression is associated with reduced breastfeeding (Seimyr et al. 2004; Pippins et al. 2006; Fairlie et al. 2009; Insaf et al. 2011; Figueiredo et al. 2014). In contrast to these studies, many of which studied EBF, our analysis investigating EBF found that depression did not predict EBF. Further, while some of these studies accounted for prenatal depression (Seimyr et al. 2004; Figueiredo et al. 2014), the discrepancy may be a result of the longitudinal nature of the current analysis. Additionally, the prevalence of EBF at 6-months postpartum was particularly low in this setting, so results may be unstable or incomparable.

Our research supports recommendations to attempt to breastfeed (WHO 2018; Meek et al. 2022) and the continuation of the Lady Health Worker Program established in 1994 to improve quality of health services in rural settings in Pakistan (Zhu et al. 2014). This program includes the incorporation of breastfeeding education programs during pregnancy to help address negative perceptions and alleviate anxieties about breastfeeding before the postpartum period. For postpartum individuals struggling or unhappy with breastfeeding, additional support from the Lady Health Workers may be beneficial. This support could include hand and pump expression support, management of breast conditions like plugged ducts, and home and community-based counseling to educate families and provide a buffer against contrary feeding advice from family members (Singletary and Farooqi 2024). For perinatally depressed individuals these supports could extend to include cognitive behavioral therapy-based interventions (Rahman et al. 2008).

### Strengths and limitations

This study uses longitudinal data spanning pregnancy to 12-months postpartum to unpack the complex relationship between perinatal depression and breastfeeding. The nature of the data and our modeling techniques allowed us to estimate the causal impacts. Further, depression was assessed using the SCID, a robust, diagnostic approach. Despite its many strengths, the study has a few limitations. While we could control for often overlooked confounders like social support and IPV, we were not able to control for anxiety symptoms or breastfeeding difficulties which may affect the relationship between breastfeeding and PPD (Pope and Mazmanian 2016). Furthermore, breastfeeding intention and support may be important modifiers (Lubis et al. 2024), but these data were not available. Both breastfeeding and depression were maintained as binary. Dichotomizing both depression and breastfeeding may have obscured variations in the severity of depression or the timing of integration of solid foods. Last, some estimates were unstable due to small sample sizes.

## Conclusions

In this longitudinal study of perinatal people in rural Pakistan, any breastfeeding was found to be moderately protective for PPD, regardless of prenatal depression. Perinatal depression also significantly reduced the likelihood of any breastfeeding. EBF was not as protective for PPD, particularly for those with prenatal depression. More work investigating this relationship in LMIC settings using longitudinal data and robust measures of depression are needed. It will be important to consider other factors such as the role of anxiety and breastfeeding intention and efficacy.

**Table 1:** Maternal characteristics and key study variables (n=1,014) prior to multiple imputation

	Mean^a^	SD^a^
Age at delivery of index child	26.5	5.4
Years of education	8.1	5.5
Socioeconomic status^b^	0.2	1.9
Parity	1.4	1.7
Perceived social support^c^	4.6	1.5
	N^a^	%^a^
Household structure		
Nuclear family	131	12.0
Non-nuclear family	883	88.0
History of intimate partner violence^d^		
Yes	480	45.8
No	481	54.2
Missing	53	
Observed chilla		
Yes	786	89.6
No	99	10.4
Missing	129	
Any breastfeeding^f^ during…		
Delivery (*n* = 885)	654	73.6
3-months (*n* = 885)	822	93.1
6-months (*n* = 929)	795	86.1
12-months (*n* = 940)	685	73.0
Exclusive breastfeeding^g^ during…		
Delivery (*n* = 885)	497	56.1
3-months (*n* = 885)	430	49.3
6-months (*n* = 929)	81	8.7
Depression^h^ during…		
Third trimester (*n* = 1,014)	372	25.1
3-months (*n* = 885)	135	13.1
6-months (*n* = 926)	120	10.8
12-months (*n* = 938)	175	15.7

^a^ Percentages, means, and standard deviations are weighted using sampling weights, Ns are unweighted

^b^ Summary of assets calculated using polychoric PCA (ranges − 5 to 2.8)

^c^ Measured with the Multidimensional Scale of Perceived Social Support (MSPSS)

^d^ Measured with the WHO Violence Against Women Instrument

^e^ Measured with the Maternal Social Support Index (MSSI) item measuring the number of tasks done alone

^f^ In past 24 h, infant given breastmilk, full description included in Appendix I

^g^ In past 24 h, infant *only* given breastmilk, with the exception of ghutti at delivery, full description included in Appendix I

^h^ Using the Structured Clinical Interview for DSM Disorders (SCID)

**Table 2:** The longitudinal relationship^a^ between *any* breastfeeding^b^ and depression^c^ from pregnancy to 12-months postpartum using marginal structural modeling on a multiply imputed dataset (n=1,014)

	RR	95% CI
Any breastfeeding ➔ Depression		
Overall	0.77	0.51–1.03
Depressed at baseline	0.74	0.44–1.05
Not depressed at baseline	0.80	0.40–1.21
Depression ➔ Any breastfeeding	**0.92**	**0.84–0.99**

RR: Risk ratio; CI: Confidence Interval

^a^ Multiple imputation by chained equations used to account for loss-to-follow-up; inverse probability of treatment weighting used to account for confounding and the role of prior treatment and outcome status (e.g. prior breastfeeding/depression)

^b^ In past 24 h, infant given breastmilk, full description included in Appendix I

^c^ Using the Structured Clinical Interview for DSM Disorders (SCID)

**Table 3:** The longitudinal relationship^a^ between *exclusive* breastfeeding^b^ and depression^c^ from pregnancy to 6-months postpartum using marginal structural modeling on a multiply imputed dataset (n=1,014)

	RR	95% CI
Exclusive breastfeeding ➔ Depression		
Overall	0.94	0.68-1.20
Depressed at baseline	1.03	0.71–1.34
Not depressed at baseline	0.77	0.31–1.23
Depression ➔ Exclusive breastfeeding	1.19	0.99–1.39

RR: Risk ratio; CI: Confidence Interval

^a^ Multiple imputation by chained equations used to account for loss-to-follow-up; inverse probability of treatment weighting used to account for confounding and the role of prior treatment and outcome status (e.g. prior breastfeeding/depression)

^b^ In past 24 h, infant *only* given breastmilk, with the exception of cultural practices at delivery, full description included in Appendix I

^c^ Using the Structured Clinical Interview for DSM Disorders (SCID)

## Data Availability

Bachpan data for the first three waves are available in the Harvard Dataverse (https://doi.org/10.7910/DVN/IJE2PC); additional data are available upon reasonable request from the senior author.

## Appendix I

**Table 4:** Infant feeding responses and categorization for analysis

	Infant feeding responses									Categorization			
**Time**	Colostrum or breastmilk	Ghutti	Herbal water (kehwa, gripe water)	Water	Tea	Formula milk	Other animal milk	Semi-solid food^a^	Solid food^a^	Any	Exclusive	Partial	None
Delivery	X							---	---	X	X		
	X	X						---	---	X	X		
	X		X					---	---	X	X		
	X	X	X					---	---	X	X		
	X	X	X	X		X		---	---	X		X	
	X	X	X			X		---	---	X		X	
	X	X	X				X	---	---	X		X	
	X	X		X		X		---	---	X		X	
	X	X		X				---	---	X		X	
	X	X			X			---	---	X		X	
	X	X				X		---	---	X		X	
	X	X					X	---	---	X		X	
	X		X			X		---	---	X		X	
	X			X		X		---	---	X		X	
	X			X				---	---	X		X	
	X					X		---	---	X		X	
	X						X	---	---	X		X	
		X	X			X		---	---				X
		X	X				X	---	---				X
		X	X					---	---				X
		X		X				---	---				X
		X			X			---	---				X
		X				X	X	---	---				X
		X				X		---	---				X
		X					X	---	---				X
		X						---	---				X
			X			X		---	---				X
			X					---	---				X
					X			---	---				X
						X		---	---				X
							X	---	---				X
								---	---				X
3-months and beyond	X									X	X		
	X								X	X		X	
	X							X		X		X	
	X						X			X		X	
	X						X	X		X		X	
	X					X				X		X	
	X					X			X	X		X	
	X					X	X			X		X	
	X					X	X	X		X		X	
	X				X					X		X	
	X				X		X			X		X	
	X				X	X				X		X	
	X				X	X	X			X		X	
	X			X						X		X	
	X			X			X			X		X	
	X			X			X	X		X		X	
	X			X			X	X	X	X		X	
	X			X		X				X		X	
	X			X	X					X		X	
	X			X	X		X			X		X	
	X			X	X	X				X		X	
	X		X							X		X	
	X		X						X	X		X	
	X		X					X		X		X	
	X		X					X	X	X		X	
	X		X				X			X		X	
	X		X				X		X	X		X	
	X		X				X	X		X		X	
	X		X				X	X	X	X		X	
	X		X			X				X		X	
	X		X			X		X		X		X	
	X		X			X		X	X	X		X	
	X		X			X	X			X		X	
	X		X			X	X	X		X		X	
	X		X		X					X		X	
	X		X		X		X			X		X	
	X		X		X		X		X	X		X	
	X		X		X		X	X		X		X	
	X		X		X	X				X		X	
	X		X	X						X		X	
	X		X	X					X	X		X	
	X		X	X				X		X		X	
	X		X	X			X			X		X	
	X		X	X			X	X		X		X	
	X		X	X		X				X		X	
	X		X	X		X			X	X		X	
	X		X	X		X		X		X		X	
	X		X	X		X	X			X		X	
	X		X	X	X		X			X		X	
	X		X	X	X	X	X			X		X	
	X	X								X		X	
	X	X					X			X		X	
	X	X					X		X	X		X	
	X	X					X	X		X		X	
	X	X				X				X		X	
	X	X				X	X			X		X	
	X	X				X	X	X		X		X	
	X	X			X					X		X	
	X	X			X		X			X		X	
	X	X			X		X	X		X		X	
	X	X		X						X		X	
	X	X		X				X		X		X	
	X	X		X			X			X		X	
	X	X		X		X				X		X	
	X	X		X		X	X			X		X	
	X	X	X							X		X	
	X	X	X					X		X		X	
	X	X	X					X	X	X		X	
	X	X	X				X			X		X	
	X	X	X				X	X		X		X	
	X	X	X			X				X		X	
	X	X	X			X			X	X		X	
	X	X	X			X	X			X		X	
	X	X	X			X	X		X	X		X	
	X	X	X			X	X	X		X		X	
	X	X	X		X					X		X	
	X	X	X		X				X	X		X	
	X	X	X		X		X			X		X	
	X	X	X		X		X		X	X		X	
	X	X	X		X		X	X		X		X	
	X	X	X		X	X	X			X		X	
	X	X	X	X						X		X	
	X	X	X	X			X			X		X	
	X	X	X	X			X	X		X		X	
	X	X	X	X		X				X		X	
	X	X	X	X		X			X	X		X	
	X	X	X	X		X		X		X		X	
	X	X	X	X		X	X			X		X	
	X	X	X	X	X		X			X		X	
		X	X	X		X	X						X
		X	X		X	X	X						X
		X	X		X	X							X
		X	X		X		X						X
		X	X		X								X
		X	X			X	X	X					X
		X	X			X	X		X				X
		X	X			X	X						X
		X	X			X							X
		X		X		X							X
		X			X		X						X
		X				X	X						X
		X				X		X					X
		X				X			X				X
		X				X							X
		X					X	X					X
			X	X		X	X						X
			X		X	X	X		X				X
			X		X	X	X						X
			X		X		X	X					X
			X		X		X						X
			X		X								X
			X			X	X	X					X
			X			X	X		X				X
			X			X	X						X
			X			X		X					X
			X			X							X
			X				X						X
					X	X	X						X
					X		X						X
					X								X
						X	X						X
						X			X				X
						X							X

^a^ Only asked at 3-months and beyond

## Appendix II

**Table 5:** The longitudinal relationship^a^ between any breastfeeding^b^ and depression^c^ from pregnancy to 6-months postpartum using marginal structural modeling on a multiply imputed dataset (n = 1,014)

	RR	95% CI
Any breastfeeding ➔Depression		
Overall	0.67	0.28–1.06
Depressed at baseline	0.74	0.24–1.23
Not depressed at baseline	0.77	0.31–1.23
Depression ➔ Any breastfeeding	**0.92**	**0.84–0.99**

RR: Risk ratio; CI: Confidence Interval

^a^ Multiple imputation by chained equations used to account for loss-to-follow-up; inverse probability of treatment weighting used to account for confounding and the role of prior treatment and outcome status (e.g. prior breastfeeding/depression)

^b^ In past 24 h, infant given breastmilk, full description included in Appendix I

^c^ Using the Structured Clinical Interview for DSM Disorders (SCID)

## References

[CR3] Agler RA, Zivich PN, Kawende B et al (2021) Postpartum depressive symptoms following implementation of the 10 steps to successful breastfeeding program in Kinshasa, Democratic Republic of Congo: a cohort study. PLoS Med 18:e1003465. 10.1371/journal.pmed.1003465 33428617 PMC7799755

[CR4] Ahlqvist-Björkroth S, Vaarno J, Junttila N et al (2016) Initiation and exclusivity of breastfeeding: association with mothers’ and fathers’ prenatal and postnatal depression and marital distress. Acta Obstet Gynecol Scand 95:396–404. 10.1111/aogs.12857 26826608

[CR5] Alimi R, Azmoude E, Moradi M, Zamani M (2022) The association of breastfeeding with a reduced risk of postpartum depression: a systematic review and meta-analysis. Breastfeed Med 17:290–296. 10.1089/bfm.2021.0183 34964664

[CR6] Asim M, Ahmed ZH, Hayward MD, Widen EM (2020) Prelacteal feeding practices in Pakistan: a mixed-methods study. Int Breastfeed J 15:53. 10.1186/s13006-020-00295-8 32513203 PMC7278149

[CR7] Azur MJ, Stuart EA, Frangakis C, Leaf PJ (2011) Multiple imputation by chained equations: what is it and how does it work? Int J Methods Psychiatr Res 20:40–49. 10.1002/mpr.329 21499542 PMC3074241

[CR8] Bogen DL, Hanusa BH, Moses-Kolko E, Wisner KL (2010) Are maternal depression or symptom severity associated with breastfeeding intention or outcomes? J Clin Psychiatry 71:1069–1078. 10.4088/jcp.09m05383blu 20584521 PMC4426491

[CR9] Brion M-J, Lawlor DA, Matijasevich A et al (2011) What are the causal effects of breastfeeding on IQ, obesity and blood pressure? Evidence from comparing high-income with middle-income cohorts. Int J Epidemiol 40:670–680. 10.1093/ije/dyr020 21349903 PMC3147072

[CR10] Bruce KE, Wouk K, Grewen KM et al (2025) HPA axis dysregulation and postpartum depression and anxiety symptoms in breastfeeding vs bottle-feeding parents. Psychoneuroendocrinology 172:107253. 10.1016/j.psyneuen.2024.107253 39675161 PMC11830542

[CR11] Butler MS, Young SL, Tuthill EL (2021) Perinatal depressive symptoms and breastfeeding behaviors: a systematic literature review and biosocial research agenda. J Affect Disord 283:441–471. 10.1016/j.jad.2020.11.080 33272686 PMC7954873

[CR12] Castro RTA, Glover V, Ehlert U, O’Connor TG (2017) Antenatal psychological and socioeconomic predictors of breastfeeding in a large community sample. Early Hum Dev 110:50–56. 10.1016/j.earlhumdev.2017.04.010 28595128

[CR14] Cooke M, Schmied V, Sheehan A (2007) An exploration of the relationship between postnatal distress and maternal role attainment, breast feeding problems and breast feeding cessation in Australia. Midwifery 23:66–76. 10.1016/j.midw.2005.12.003 17011682

[CR13] Coo S, García MI, Mira A, Valdés V (2020) The role of perinatal anxiety and depression in breastfeeding practices. Breastfeed Med 15:495–500. 10.1089/bfm.2020.0091 32522015

[CR15] Dennis C-L (1999) Theoretical underpinnings of breastfeeding confidence: a self-efficacy framework. J Hum Lact 15:195–201. 10.1177/089033449901500303 10578797

[CR16] Dennis CL, McQueen K (2007) Does maternal postpartum depressive symptomatology influence infant feeding outcomes? Acta Paediatr 96:590–594. 10.1111/j.1651-2227.2007.00184.x 17391475

[CR17] Dias CC, Figueiredo B (2015) Breastfeeding and depression: a systematic review of the literature. J Affect Disord 171:142–154. 10.1016/j.jad.2014.09.022 25305429

[CR18] Ding T, Wang D-X, Qu Y et al (2014) Epidural labor analgesia is associated with a decreased risk of postpartum depression: a prospective cohort study. Anesth Analg 119:383–392. 10.1213/ane.0000000000000107 24797120

[CR19] Duijts L, Jaddoe VWV, Hofman A, Moll HA (2010) Prolonged and exclusive breastfeeding reduces the risk of infectious diseases in infancy. Pediatrics 126:e18–e25. 10.1542/peds.2008-3256 20566605

[CR20] Ebina S, Kashiwakura I (2012) Influence of breastfeeding on maternal blood pressure at one month postpartum. Int J Womens Health 4:333–339. 10.2147/ijwh.s33379 22870047 PMC3410704

[CR21] Fairlie TG, Gillman MW, Rich-Edwards J (2009) High pregnancy-related anxiety and prenatal depressive symptoms as predictors of intention to breastfeed and breastfeeding initiation. J Womens Health 18:945–953. 10.1089/jwh.2008.0998 PMC2851128 19563244

[CR22] Farías-Antúnez S, Santos IS, Matijasevich A, de Barros AJD (2020) Maternal mood symptoms in pregnancy and postpartum depression: association with exclusive breastfeeding in a population-based birth cohort. Soc Psychiatry Psychiatr Epidemiol 55:635–643. 10.1007/s00127-019-01827-2 31897581

[CR24] Figueiredo B, Canário C, Field T (2014) Breastfeeding is negatively affected by prenatal depression and reduces postpartum depression. Psychol Med 44:927–936. 10.1017/s0033291713001530 23822932

[CR23] Figueiredo B, Dias CC, Brandão S et al (2013) Breastfeeding and postpartum depression: state of the art review. J Pediatr 89:332–338. 10.1016/j.jped.2012.12.002 23791236

[CR25] Gagliardi L, Petrozzi A, Rusconi F (2012) Symptoms of maternal depression immediately after delivery predict unsuccessful breast feeding. Arch Dis Child 97:355. 10.1136/adc.2009.179697 21127006

[CR26] Gallis J, Sanborn K, Turner E (2024) Bachpan Study of Maternal Depression and Child Development: Data from the First Three Years. In: Harvard Dataverse, VI. 10.7910/DVN/IJE2PC

[CR27] González-Jiménez E, García PA, Aguilar MJ et al (2014) Breastfeeding and the prevention of breast cancer: a retrospective review of clinical histories. J Clin Nurs 23:2397–2403. 10.1111/jocn.12368 23937211

[CR28] Gorman LL, O’Hara MW, Figueiredo B et al (2004) Adaptation of the Structured Clinical Interview for DSM-IV Disorders for assessing depression in women during pregnancy and post-partum across countries and cultures. Br J Psychiatry 184:s17–s23. 10.1192/bjp.184.46.s17 14754814

[CR29] Haga SM, Lisøy C, Drozd F et al (2018) A population-based study of the relationship between perinatal depressive symptoms and breastfeeding: a cross-lagged panel study. Arch Women’s Ment Heal 21:235–242. 10.1007/s00737-017-0792-z 29063201

[CR30] Hahn-Holbrook J, Haselton MG, Schetter CD, Glynn LM (2013) Does breastfeeding offer protection against maternal depressive symptomatology? Arch Women’s Ment Heal 16:411–422. 10.1007/s00737-013-0348-9 PMC3818091 23749095

[CR31] Hamdan A, Tamim H (2011) Psychosocial risk and protective factors for postpartum depression in the United Arab Emirates. Arch Women’s Ment Heal 14:125–133. 10.1007/s00737-010-0189-8 21063891

[CR32] Hamdan A, Tamim H (2012) The relationship between postpartum depression and breastfeeding. Int J Psychiatry Med 43:243–259. 10.2190/pm.43.3.d 22978082

[CR33] Hasselmann MH, Werneck GL, da Silva CVC (2008) Symptoms of postpartum depression and early interruption of exclusive breastfeeding in the first two months of life. Cad Saude Publica 24:s341–s352. 10.1590/s0102-311x2008001400019 18670714

[CR34] Henderson JJ, Evans SF, Straton JAY et al (2003) Impact of postnatal depression on breastfeeding duration. Birth 30:175–180. 10.1046/j.1523-536x.2003.00242.x 12911800

[CR35] Horta BL, de Mola CL, Victora CG (2015) Long-term consequences of breastfeeding on cholesterol, obesity, systolic blood pressure and type 2 diabetes: a systematic review and meta‐analysis. Acta Paediatr 104:30–37. 10.1111/apa.13133 26192560

[CR36] Insaf TZ, Fortner RT, Pekow P et al (2011) Prenatal stress, anxiety, and depressive symptoms as predictors of intention to breastfeed among Hispanic women. J Womens Health Larchmt 20:1183–1192. 10.1089/jwh.2010.2276 21668379

[CR37] Jain A, Tyagi P, Kaur P et al (2014) Association of birth of girls with postnatal depression and exclusive breastfeeding: an observational study. BMJ Open 4:e003545. 10.1136/bmjopen-2013-003545 24913326 PMC4054658

[CR38] Kolenikov S, Angeles G (2009) Socioeconomic status measurement with discrete proxy variables: is principal component analysis a reliable answer? Rev Income Wealth 55:128–165. 10.1111/j.1475-4991.2008.00309.x

[CR39] Lubis PN, Saputra M, Rabbani MW (2024) A systematic review of the benefits of breastfeeding against postpartum depression in low-middle-income countries. J Ment Heal ahead-of-print 1–13. 10.1080/09638237.2024.2361232 38869015

[CR40] Maselko J, Bates L, Bhalotra S et al (2018) Socioeconomic status indicators and common mental disorders: evidence from a study of prenatal depression in Pakistan. SSM Popul Health 4:1–9. 10.1016/j.ssmph.2017.10.004 29349268 PMC5769091

[CR78] Maselko J, Sikander S, Turner EL et al (2020) Effectiveness of a peer-delivered, psychosocial intervention on maternal depression and child development at 3 years postnatal: a cluster randomised trial in Pakistan. Lancet Psychiatry 7:775–87. 10.1016/S2215-0366(20)30258-3 PMC8015797 32828167

[CR41] McCarter-Spaulding D, Horowitz JA (2007) How does postpartum depression affect breastfeeding? MCN Am J Matern Child Nurs 1:10–17 10.1097/00005721-200701000-00004 17308452

[CR42] Meek JY, Noble L, Breastfeeding S on (2022) Policy statement: breastfeeding and the use of human milk. Pediatrics. 10.1542/peds.2022-057988 35921640

[CR43] Mezzacappa ES, Endicott J (2007) Parity mediates the association between infant feeding method and maternal depressive symptoms in the postpartum. Arch Womens Ment Health 10:259–266. 10.1007/s00737-007-0207-7 18040595

[CR44] Nast I, Bolten M, Meinlschmidt G, Hellhammer DH (2013) How to measure prenatal stress? A systematic review of psychometric instruments to assess psychosocial stress during pregnancy. Paediatr Perinat Epidemiol 27:313–322. 10.1111/ppe.12051 23772932

[CR45] National Institute of Population Studies Islamabad Pakistan (2019) Pakistan demographic and health survey (DHS), 2017-18 - Final report (English)

[CR46] Nazel MWA, Nosseir SA (1994) Antepartum and postpartum depression and infant feeding pattern: a prospective study. J Egypt Public Health Assoc 69:397–424 17212007

[CR47] Pippins JR, Brawarsky P, Jackson RA et al (2006) Association of breastfeeding with maternal depressive symptoms. J Womens Health Larchmt 15:754–762. 10.1089/jwh.2006.15.754 16910907

[CR48] Pope CJ, Mazmanian D (2016) Breastfeeding and postpartum depression: an overview and methodological recommendations for future research. Depress Res Treat 2016:4765310. 10.1155/2016/4765310 27148457 PMC4842365

[CR51] Rahman A, Hafeez A, Bilal R et al (2016) Impact of perinatal depression on breastfeeding. Maternal Child Nutr 12:452–462. 10.1111/mcn.12170 PMC6860115 25682731

[CR49] Rahman A, Iqbal Z, Waheed W, Hussain N (2003) Translation and cultural adaptation of health questionnaires. JPMA J Pak Med Assoc 53:142–147 12776898

[CR50] Rahman A, Malik A, Sikander S et al (2008) Cognitive behaviour therapy-based intervention by community health workers for mothers with depression and their infants in rural Pakistan: a cluster-randomised controlled trial. Lancet 372:902–909. 10.1016/s0140-6736(08)61400-2 18790313 PMC2603063

[CR52] Rehman U, Atiq (2016) The relationship between breastfeeding, sleep, and postpartum depression. J Soc Obstetricians Gynecologists Pakistan 6:119–124

[CR53] Robins JM, Hernán MÁ, Brumback B (2000) Marginal structural models and causal inference in epidemiology. Epidemiology 11:550–560. 10.1097/00001648-200009000-00011 10955408

[CR54] Rollins NC, Bhandari N, Hajeebhoy N et al (2016) Why invest, and what it will take to improve breastfeeding practices? Lancet 387:491–504. 10.1016/s0140-6736(15)01044-2 26869576

[CR55] Seimyr L, Edhborg M, Lundh W, Sjögren B (2004) In the shadow of maternal depressed mood: experiences of parenthood during the first year after childbirth. J Psychosom Obstet Gynecol 25:23–34. 10.1080/01674820410001737414 15376402

[CR56] Shah S, Lonergan B (2017) Frequency of postpartum depression and its association with breastfeeding: a cross-sectional survey at immunization clinics in Islamabad, Pakistan. JPMA J Pak Méd Assoc 67:1151–1156 28839296

[CR57] Sharifi F, Nouraei S, Shahverdi E (2016) The relation of pre and postnatal depression and anxiety with exclusive breastfeeding. Electron physician 8:3234–3239. 10.19082/3234 28070257 PMC5217816

[CR77] Sikander S, Ahmad I, Bates LM et al (2019) Cohort Profile: Perinatal depression and child socioemotional development; the Bachpan cohort study from rural Pakistan. BMJ open 9:e025644. 10.1136/bmjopen-2018-025644 PMC6502044 31061029

[CR58] Singletary N, Farooqi ZW (2024) Beliefs, experiences, and practices of Lady Health Workers in facilitating breastfeeding in rural communities in Pakistan. Qual Heal Res 34:1339–1350. 10.1177/10497323241242640 38830234

[CR59] Spitzer RL, Williams JBW, Gibbon M, First MB (1992) The structured clinical interview for DSM-III-R (SCID): I: history, rationale, and description. Arch Gen Psychiatry 49:624–629. 10.1001/archpsyc.1992.01820080032005 1637252

[CR60] Stuebe AM, Grewen K, Meltzer-Brody S (2013) Association between maternal mood and oxytocin response to breastfeeding. Journal of Women’s Health 22:352–361. 10.1089/jwh.2012.3768 23586800 PMC3627433

[CR61] Taj R, Sikander KS (2003) Effects of maternal depression on breast-feeding. JPMA J Pak Méd Assoc 53:8–11 12666844

[CR62] Toledo C, Cianelli R, Rodriguez NV et al (2022) The significance of breastfeeding practices on postpartum depression risk. Public Health Nurs 39:15–23. 10.1111/phn.12969 34510526

[CR63] Tschiderer L, Seekircher L, Kunutsor SK et al (2021) J Am Hear Assoc: Cardiovasc Cerebrovasc Dis 11:e022746. 10.1161/jaha.121.022746. Breastfeeding Is Associated With a Reduced Maternal Cardiovascular Risk: Systematic Review and Meta-Analysis Involving Data From 8 Studies and 1 192 700 Parous Women PMC9238515 35014854

[CR64] Tuthill EL, Pellowski JA, Young SL, Butler LM (2017) Perinatal depression among HIV-Infected women in KwaZulu-Natal South Africa: Prenatal depression predicts lower rates of exclusive breastfeeding. AIDS Behav 21:1691–1698. 10.1007/s10461-016-1557-9 27752868 PMC5393963

[CR65] Whitley J, Wouk K, Bauer AE et al (2020) Oxytocin during breastfeeding and maternal mood symptoms. Psychoneuroendocrinology 113:104581. 10.1016/j.psyneuen.2019.104581 31911347 PMC8117182

[CR1] (WHO) undefined WHO (2005) WHO multi-country study on women’s health and domestic violence against women

[CR2] (WHO) WHO (2018) Guideline: Counseling of women to improve breastfeeding practices. World Health Organization, Geneva 30933442

[CR66] Wulff JN, Jeppesen LE (2017) Multiple imputation by chained equations in praxis: Guidelines and review. Electron J Bus Res Methods 1:41–56

[CR67] Yahya NFS, Teng NIMF, Shafiee N, Juliana N (2021) Association between breastfeeding attitudes and postpartum depression among mothers with premature infants during COVID-19 pandemic. Int J Environ Res Public Health 18:10915. 10.3390/ijerph182010915 34682652 PMC8535779

[CR68] Ystrom E (2012) Breastfeeding cessation and symptoms of anxiety and depression: A longitudinal cohort study. BMC Pregnancy Childbirth 12:36. 10.1186/1471-2393-12-36 22621668 PMC3449190

[CR69] Yuen M, Hall OJ, Masters GA et al (2022) The effects of breastfeeding on maternal mental health: A systematic review. J Womens Health Larchmt 31:787–807. 10.1089/jwh.2021.0504 35442804

[CR70] Yusuff ASM, Tang L, Binns CW, Lee AH (2016a) Prevalence of antenatal depressive symptoms among women in Sabah, Malaysia. J Matern Fetal Neonatal Med 29:1170–1174. 10.3109/14767058.2015.1039506 26037724

[CR71] Yusuff ASM, Tang L, Binns CW, Lee AH (2016b) Breastfeeding and postnatal depression: A prospective cohort study in Sabah, Malaysia. J Hum Lact 32:277–81. 10.1177/0890334415620788 26644418

[CR72] Zakar R, Zakar MZ, Zaheer L, Fischer F (2018) Exploring parental perceptions and knowledge regarding breastfeeding practices in Rajanpur, Punjab Province, Pakistan. Int Breastfeed J 13:24. 10.1186/s13006-018-0171-z 29988704 PMC6029391

[CR73] Zhu N, Allen E, Kearns A et al (2014) Lady Health Workers in Pakistan

[CR74] Zimet GD, Dahlem NW, Zimet SG, Farley GK (1988) The multidimensional scale of perceived social support. J Pers Assess 52:30–41. 10.1207/s15327752jpa5201_2 2280326

[CR75] Zimet GD, Powell SS, Farley GK et al (1990) Psychometric characteristics of the multidimensional scale of perceived social support. J Pers Assess 55:610–617. 10.1080/00223891.1990.9674095 2280326

[CR76] Zong X, Wu H, Zhao M et al (2021) Global prevalence of WHO infant feeding practices in 57 LMICs in 2010–2018 and time trends since 2000 for 44 LMICs. EClinicalMedicine 37:100971. 10.1016/j.eclinm.2021.100971 34386748 PMC8343261

